# The first study appraising colonic diverticulosis and *Helicobacter pylori* diagnosed by histopathology

**DOI:** 10.1590/1806-9282.20240400

**Published:** 2024-07-19

**Authors:** Ersin Kuloglu, Kubilay Issever, Ali Muhtaroglu, Gokhan Aydın, Sefer Aslan, Aykut Ozturan, Demet Sengul, Esma Cinar, Ahmet Cumhur Dulger, Ilker Sengul

**Affiliations:** 1 Giresun University, Faculty of Medicine, Department of Internal Medicine – Giresun, Turkey.; 2 Giresun University, Faculty of Medicine, Department of General Surgery – Giresun, Turkey.; 3 Hatay Iskenderun State Hospital, Department of Gastroenterology – Hatay, Turkey.; 4 Giresun Education and Research Hospital, Department of Internal Medicine – Giresun, Turkey.; 5 Giresun University, Faculty of Medicine, Department of Pathology – Giresun, Turkey.; 6 Giresun University, Faculty of Medicine, Division of Gastroenterology – Giresun, Turkey.; 7 Giresun University, Faculty of Medicine, Division of Endocrine Surgery – Giresun, Turkey.

**Keywords:** Diverticulum, Helicobacter pylori, Gastric mucosa, Metaplasia, Pathology

## Abstract

**OBJECTIVE:**

Colonic diverticulosis might be caused by low-fiber dietary habits, gastrointestinal motility disorders, and colonic wall resistance changes, which might also affect the upper gastrointestinal system mucosa. Therefore, the present study aims to answer whether the gastric histopathological findings of the cases with diverge from those without.

**METHODS:**

This retrospective cross-sectional study included 184 cases who underwent both upper and lower gastrointestinal endoscopy procedures between January 2020 and December 2022. Notably, 84 cases were colonic diverticulosis, while the rest of the study group was control. Their demographic, laboratory, and histopathological findings were compared meticulously.

**RESULTS:**

The median ages for the colonic diverticulosis and control were 67.07±8.14 and 66.29±15.83 years, respectively, and no statistical difference concerning the age and gender distribution between them was recognized. The median levels of white blood cells, neutrophils, glucose, creatinine, and aspartate aminotransferase in colonic diverticulosis were significantly increased compared to control. As for pathological comparison, colonic diverticulosis had a higher prevalence of *Helicobacter pylori* (45.2 vs. 38%), while atrophy and intestinal metaplasia prevalence were nearly the same in the groups, without significance regarding *Helicobacter pylori*.

**CONCLUSION:**

Consequently, colonic diverticulosis should not be overlooked, particularly when the abovementioned laboratory parameters are augmented in a dyspeptic patient. A correlation might be raised between *Helicobacter pylori* and colonic diverticulosis. Eradication therapy might help attenuate the risk of colonic diverticulosis when *Helicobacter pylori* has emerged in a patient.

## INTRODUCTION

Colonic diverticulosis (CoDiv) is characterized by the herniation of the mucosa through weaknesses in the muscle layer due to insufficiency. Diverticulosis commonly occurs when blood vessels penetrate the muscle layer, weakening those areas for diverticula formation, which are frequently found in the distal colon, with approximately 90% of patients exhibiting diverticula in the sigmoid colon in the continents of Europe and USA^1^. The prevalence of diverticulosis increases with age and is observed in about 50% of individuals aged 60 years in Western societies^2^. The prevalence of CoDiv varies from under 10% in individuals aged under 40 years to 50–66% in the geriatric population aged over 80 years^3^. Of note, CoDiv can be asymptomatic. However, 5–20% of the cases experience recurrent abdominal pain, gastrointestinal (GI) bleeding, and diverticulitis^4^. It is highly prevalent, with increased incidence and prevalence alongside rising socioeconomic standards in developed countries^5^, and its development in Western societies has been linked to a diet low in fiber and roughage^6^. Along with a low-fiber diet, changes in colon wall resistance and colonic motility disorders are the most widely accepted etiological factors^6^. In addition, constipation, lack of physical activity, smoking, use of nonsteroidal anti-inflammatory drugs, and inflammation are thought to play roles in the occurrence of this phenomenon^7^. Moreover, male gender, alcohol consumption, prediabetic conditions, increased serum triglyceride levels, gut microbiota alterations, and obesity are asserted with diverticula formation^8,9^. Although genetic factors and ethnic background play a role in the etiology of CoDiv, its impact is considered less significant than lifestyle and dietary habits^10^.

The development of CoDiv and HP infection is related to socioeconomic status, and their prevalences increase with age. Furthermore, *Helicobacter pylori* (*H. pylori*) might cause mucosal changes in the upper GI system, such as atrophic gastritis and intestinal metaplasia^11^, which leads us to think that patients with *H. pylori* infection and CoDiv might have some typical characterizations that facere them more prone to mucosal changes in the GI system. However, to the best of our knowledge, a study regarding the relationship between *H. pylori* and CoDiv has been recognized in the English-language literature^5^. Therefore, our study aimed to evaluate the gastric histopathological findings of the patients with CoDiv, establish possible associations with *H. pylori* infection, and compare them with patients without the diverticula.

## METHODS

### Study design

The present study was conducted according to the Declaration of Helsinki. This retrospective cross-sectional study was conducted at the Giresun Education and Research Hospital, Giresun, Turkey, from January 2020 to December 2022. The study incorporated a total of 184 cases who had undergone upper and lower GI endoscopy procedures. Of these, 84 cases had been diagnosed with pancolonic diverticulosis, while a control of 100 showed similar demographic characteristics without possessing diverticulosis. CoDiv diagnoses were provided through endoscopic examination characterized by the detection of pouches extending from the colonic wall, and the details, such as the location, size, and appearance of the diverticula, were recorded. The control comprised cases with similar symptoms without colonic diverticulum in the endoscopy. All the procedures were performed by a unique gastroenterologist. The demographic data, such as age, sex, and co-morbidities of the cases, were obtained from the digital medical records of the hospital.

### Laboratory parameters

All the cases had been examined with white blood cell count (WBC), neutrophils, hemoglobin (Hgb), mean corpuscular volume (MCV), platelet count (Plt), glucose, alanine aminotransferase (ALT), aspartate aminotransferase (AST), calcium (Ca), lymphocytes (Lymp), creatinine (Cre), sodium (Na), and potassium (K), which were recorded meticulously.

### Endoscopic procedures

**Figure 1:**
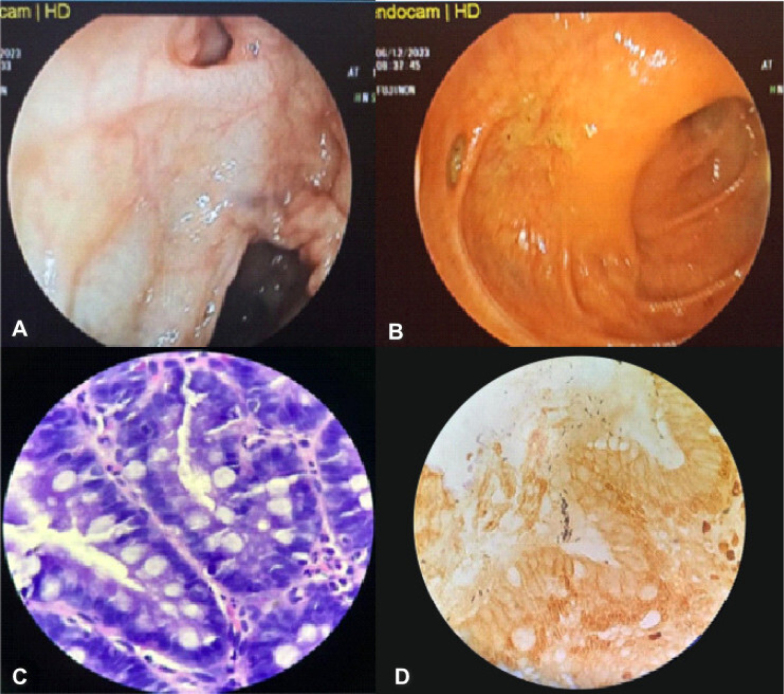
(A) and (B) The endoscopic photographs exhibiting diverticula identified in different colon segments diagnosed with colonic diverticulosis. (C) A microphotograph revealing intestinal metaplasia in colonic diverticulosis, hematoxylin and eosin (H&E) stain, original magnification, 40×. (D) A microphotograph revealing *Helicobacter pylori* positivity in colonic diverticulosis, Warthin-Starry (WS) stain, original magnification, 40×.

The endoscopic procedures with the administration of 0.1 mg/kg Dormicum and 0.5 mg/kg Propofol for sedation purposes were performed by using the Fujinon VP-4450 HD^Ò^ device in the endoscopic unit (Figures 1A, B).

### Histopathological evaluation

The endoscopic biopsy materials were immediately fixed in 10% formalin and prepared for histopathological analysis. The evaluation of *H. pylori* presence, atrophy, and intestinal metaplasia in the gastric tissue was performed under a light microscope via hematoxylin and eosin (H&E) and Warthin-Stary (WE) stains, respectively, and documented by experienced pathologists blinded to the clinical data (Figures 1C, D).

### Statistical analysis

The data were analyzed utilizing the Statistical Package for the Social Sciences (SPSS) (IBM for Windows, v.26). The study used the Skewness-Kurtosis tests for continuous measurements in order to detect the normal distribution of the variables, for which the reference range was between ±2. Furthermore, the chi-square (Fisher's exact) test was used to compare descriptive characteristics and histopathological findings between the cases with/without pancolonic diverticulosis. For the comparison of measurements exhibiting a normal distribution, the independent-samples t-test was utilized. Meanwhile, the Mann-Whitney U analysis method was employed to compare measurements that did not demonstrate a normal distribution. Statistical significance level (a) was taken as 5% (95% confidence interval) in the calculations.

## RESULTS

In all, 184 patients undergoing endoscopic procedures and the relevant histopathological evaluations were included in the analysis. A total of 45.6% of the cases possessed CoDiv, 47.6% (40) male and 52.4% (44) female, whereas 54.4% did not have CoDiv (control), 45% (45) male and 55% (55) female. A total of 31% of CoDiv were under 65 years old with a mean age of 67.07±8.14 years, while 50% were under 65 years old with a mean age of 66.29±15.83 years in control. These outcomes indicate no significant difference in the gender and age distribution between the cases with and without diverticulosis (p>0.05). In other words, this distribution is homogeneous in both groups (Table 1).

**Table 1:** Comparison of the laboratory parameters between colonic diverticulosis and control.

Laboratory parameters	CoDiv (n=84)	Control (n=100)	p-value
	Median±SD	Median±SD	
WBC^t^	7510.1±1862.1	6957.6±31892.7	0.048*
Hemoglobin^t^	12.1±1.8	12.6±2.1	0.069
MCV^t^	85.7±6.1	85.2±8	0.885
Platelet^t^	264.72±90.66	262.70±76.78	0.798
Lymphocyte^t^	1843.8±699.8	1990.7±605.4	0.128
Neutrophil	4783.1±1594	3823.8±1040.7	0.001**
Glucose^z^	119±33	105±31.5	0.001**
Creatinine^t^	0.91±0.30	0.80±0.21	0.001**
ALT^z^	18.11±8.62	15.90±8.31	0.061
AST^t^	21.44±8.91	18.90±5.53	0.036*
Na^t^	140.82±3.40	140.46±3	0.174
K^t^	4.41±0.51	4.32±0.4	0.451
Ca^t^	9.54±0.62	9.60±0.40	0.572

* p<0.05,

** p<0.01,

SD: standart deviation,

t: independent-samples t-test,

z: Mann-Whitney U test (mean and standard deviation values of the data, as well as median, minimum, and maximum values are given). CoDiv: colonic diverticulosis.

Upon comparing the laboratory parameters, no difference in Lymp, Hgb, MCV, Plt, ALT, Na, K, and Ca values was detected (p>0.05). However, significant differences were observed in WBC, neutrophil, glucose, Cre, and AST values between patients with and without diverticulosis (p<0.05) (Table 2).

**Table 2:** Comparison of histopathological findings between colonic diverticulosis and control.

Histopathological findings		CoDiv (n=84)		Control (n=100)		p-value
		N	%	n	%	
*H. pylori*	Negative	46	54.8	62	62	0.321
	Positive	38	45.2	38	38	
Atrophy	Negative	75	89.3	90	90	0.874
	Positive	9	10.7	10	10	
Metaplasia	Negative	65	77.4	77	77	0.951
	Positive	19	22.6	23	23	

Chi-square test. CoDiv: colonic diverticulosis.

In addition, the distribution of *H. pylori*, atrophy, and metaplasia did not differ, whereas the prevalence was higher numerically in CoDiv.

## DISCUSSION

The microbial component in the gastrointestinal system is most abundant in the colon, being approximately 10^7^ times greater than in the stomach. Some studies suggest that *H. pylori* enhances resistance against human gastrointestinal infections, thereby increasing fecal microbiota diversity. Another theory posits that *H. pylori* disrupts gastric acidity, allowing microorganisms to pass the gastric barrier and reach the colon^12^. A thorough literature review revealed that only one study has examined the relationship between *H. pylori* and CoDiv. Bartels et al. involved 56,001 cases in Denmark in which patients underwent urea breath testing and were followed up for 6 years. The prevalence of *H. pylori* based on the urea breath test was determined to be 20%, and the patients infected with *H. pylori* had a lower CoDiv prevalence (0.87 vs. 1.14%, respectively, OR=0.62, 95%CI: 0.50–0.78). Furthermore, after the eradication, no statistical difference in CoDiv incidence rates was reported^5^. Contrarily, the present study revealed *H. pylori* positivity at a higher rate in cases with CoDiv than those without (45.2 vs. 38%, respectively). However, we anticipate that the difference would be substantial as the present study involves more cases, although no significant difference was revealed between the study groups. In addition, no difference was recognized between them in either intestinal metaplasia or gastric atrophy. Bartels's study identified *H. pylori* non-invasively and noted that they included all the diagnostic codes regarding CoDiv, which might display a heterogenic clinical picture. Nevertheless, our study identified *H. pylori* along with atrophy and intestinal metaplasia status of gastric mucosa from gastric pathology specimens, the gold standard diagnostic method, and presented CoDiv in elective, planned endoscopic procedures rather than mixed acute and elective cases^13^. Furthermore, we had a much smaller study group without data regarding pre- and post-*H. pylori* eradication comparisons. Finally, the designs of the studies differ as Bartels et al. conducted both a cross-sectional and historical cohort study. At the same time, we performed a retrospective cross-sectional study. Considering these differences between the studies, it should not be surprising to find contradictory results regarding the effect of *H. pylori* on CoDiv progression.

Epidemiological studies have revealed a liaison between *H. pylori* infection and some clinical conditions characterized by persistent and low-grade systemic inflammation and specific diseases such as iron deficiency anemia, idiopathic thrombocytopenic purpura, and vitamin B12 deficiency^14^. Our outcomes, revealing a higher *H. pylori* prevalence and worse laboratory findings in CoDiv, are coherent with this study. However, there are also refutatory articles in the literature. Another Danish study found a lower prevalence of Crohn's and coeliac diseases but not ulcerative colitis in *H. pylori*-positivity^15^. A review investigating the liaison between *H. pylori* infection and inflammatory bowel diseases (IBD) revealed a steady negative association between gastric *H. pylori* infection and IBD^16^. These inconsistent results, along with the result of our study, manifest that the proposed immunomodulatory effects of *H. pylori*, an infectious bacteria that might lead to mucosal inflammation, are still debated and need to be proven by large randomized controlled trials^17^.

In the present study, WBS, Neu, Glc, Cre, and AST levels were significantly higher in CoDiv. Literature reveals that low-grade chronic inflammation exists in CoDiv, which is thought to be the underlying mechanism for the risk factors of this disease, such as obesity, smoking, physical inactivity, high red meat consumption, and low-fiber diet. Acute phase reactants increase further in more severe forms of CoDiv, such as symptomatic uncomplicated diverticular disease and acute diverticulitis, in which low-grade chronic inflammation might cause leukocyte and neutrophil elevation in our patient group, although they all incidentally possessed asymptomatic diverticulosis. It was shown that components of metabolic dysregulation, such as hypertension, hyperlipidemia, and hepatosteatosis, are associated with the presence and severity of CoDiv^18-25^. Considering this fact, considering higher Glc, Cre, and AST levels in CoDiv is unsurprising. Consequently, all the laboratory analyses were compatible with the current literature on CoDiv. To the best of our knowledge, this is the first study evaluating the liaison of *H. pylori* with CoDiv via gastric biopsy analysis and the second study to investigate this association in the literature. The observed elevations in WBC, Neu, Glc, Cre, and AST levels may affect future clinical guidelines and patient management strategies. This research might open avenues for further studies investigating the pathophysiological mechanisms behind these associations and their clinical implications.

### Limitations

The limitations of the present study are its retrospective nature, with a small study group without data regarding pre- and post-*H. pylori* eradication comparisons, a lack of inquiry into patients' socioeconomic levels, dietary habits, defecation routines, physical activity levels, smoking and alcohol use, lipid profiles, and nonsteroidal anti-inflammatory drug use regarding diverticular disease.

## CONCLUSION

The outcomes of the present study reveal significant associations between pancolonic diverticulosis, elevated laboratory parameters, and *H. pylori* infection in dyspeptic patients, indicating an inflammatory and metabolic components in this condition, which challenge its traditional perception as an asymptomatic condition and highlight its potential influence on dyspeptic symptomatology. These results emphasize the need to consider pancolonic diverticulosis in the differential diagnosis of dyspepsia, advocating for a more comprehensive diagnostic approach in gastroenterology, which merits further investigation.
